# Summary of taxonomy changes ratified by the International Committee on Taxonomy of Viruses (ICTV) from the Animal DNA Viruses and Retroviruses Subcommittee, 2025

**DOI:** 10.1099/jgv.0.002113

**Published:** 2025-07-25

**Authors:** Arvind Varsani, Adly M.M. Abd-Alla, Niklas Arnberg, Kelly S. Bateman, Mária Benkő, Annie Bézier, Philippe Biagini, Jamie Bojko, Anamarija Butkovic, Marta Canuti, Vladimír Celer, Jean-Michel Drezen, Laszlo Egyed, Matthias G. Fischer, Sarah François, Benjamin Guinet, Balázs Harrach, Robert L. Harrison, Elisabeth A. Herniou, Michael Hess, Jia Hu, Johannes A. Jehle, Győző L. Kaján, Adrianna E. Kajon, Eugene V. Koonin, Simona Kraberger, Peter J. Krell, Mart Krupovic, Jens H. Kuhn, Chengfeng Lei, Matthieu Leobold, Fabrizio Maggi, Suresh K. Mittal, Hiroaki Okamoto, Tanja Opriessnig, Xiaowei Peng, Judit Pénzes, Iva I. Podgorski, Thomas S. Postler, Bergmann M. Ribeiro, Carmen San Martín, Maria Söderlund-Venermo, Xiulian Sun, András Surján, Zoltán L. Tarján, Julien Varaldi, Márton Z. Vidovszky, Göran Wadell, Hidemi Watanabe, Natalya Yutin, Monique M. van Oers, ICTV Taxonomy Summary Consortium, E.M. Adriaenssens

**Affiliations:** 1 The Biodesign Center for Fundamental and Applied Microbiomics, Center for Evolution and Medicine, School of Life Sciences, Arizona State University, Tempe, AZ, USA; 2 Joint FAO/IAEA Programme of Nuclear Techniques in Food and Agriculture, Vienna International Centre, Vienna, Austria; 3 Department of Clinical Microbiology, Umeå University, Umeå, Sweden; 4 Centre for Environment, Fisheries and Aquaculture Science (CEFAS), Weymouth, Dorset, DT4 8UB, UK; 5 HUN-REN Veterinary Medical Research Institute, Budapest, Hungary; 6 Institut de Recherche sur la Biologie de l’Insecte, UMR 7261 CNRS-Université de Tours, Tours 37200, France; 7 Equipe Biologie des Groupes Sanguins, UMR 7268 ADES, Aix-Marseille Université, CNRS, EFS, 27 Bd. Jean Moulin, 13005 Marseille, France; 8 National Horizons Centre, Teesside University, Darlington, DL1 1HG, UK; 9 Institut Pasteur, Université Paris Cité, CNRS UMR6047, Archaeal Virology Unit, Paris, France; 10 Department of Veterinary and Animal Sciences, University of Copenhagen, Frederiksberg, Denmark; 11 Faculty of Veterinary Medicine, University of Veterinary Sciences Brno, Palackeho 1946, 612 42, Brno, Czech Republic; 12 Department of Biomolecular Mechanisms, Max Planck Institute for Medical Research, Heidelberg, Germany; 13 DGIMI, Univ Montpellier, INRAE, Montpellier, France; 14 Laboratoire de Biométrie et Biologie Evolutive, UMR CNRS 5558, Université Claude Bernard Lyon 1, Villeurbanne CEDEX F-69622, France; 15 Invasive Insect Biocontrol and Behavior Laboratory, USDAARS, 10300 Baltimore Avenue, Beltsville, MD, USA; 16 University of Veterinary Medicine, Vienna, Austria; 17 Wuhan Institute of Virology, Chinese Academy of Sciences, Wuhan, PR China; 18 Institute for Biological Control, Julius Kühn-Institut, Dossenheim, Germany; 19 Lovelace Respiratory Research Institute, Albuquerque, NM, USA; 20 Computational Biology Branch, Division of Intramural Research, National Library of Medicine, National Institutes of Health, Bethesda, MD 20894, USA; 21 Department of Molecular and Cellular Biology, University of Guelph, Guelph, Canada; 22 Integrated Research Facility at Fort Detrick, National Institute of Allergy and Infectious Diseases, National Institutes of Health, Fort Detrick, Frederick, MD, USA; 23 Laboratory of Virology and Laboratories of Biosecurity, National Institute for Infectious Diseases Lazzaro Spallanzani - IRCCS, Rome, Italy; 24 Purdue University, West Lafayette, IN, USA; 25 Division of Virology, Department of Infection and Immunity, Jichi Medical University School of Medicine, 3311-1 Yakushiji, Shimotsuke-shi, Tochigi 329-0498, Japan; 26 Moredun Research Institute, Pentland Science Park, Bush Loan, Penicuik, Midlothian, EH26 0PZ, UK; 27 Institute for Quantitative Biomedicine, Rutgers University, Piscataway, USA; 28 Ruđer Bošković Institute, Zagreb, Croatia; 29 International AIDS Vaccine Initiative, Jersey City, USA; 30 Laboratory of Baculovirus, Cell Biology Department, University of Brasília, Brasília, Brazil; 31 Centro Nacional de Biotecnología (CNB-CSIC), Madrid, Spain; 32 Department of Virology, University of Helsinki, Haartmaninkatu 3, 00290, Helsinki, Finland; 33 Hokkaido University, Sapporo, Japan; 34 Laboratory of Virology, Wageningen University and Research, Wageningen, Netherlands

**Keywords:** *Abadenavirae*, *African swine fever virus*, *Alphabaculovirus alterhycuneae*, *Alphabaculovirus altermaconfiguratae*, *Alphabaculovirus pastagnalis*, *Alphabaculovirus pavitrealis*, *Alphabaculovirus spocosmioidis*, *Alphafilamentovirus*, *Alphafilamentovirus leboulardi*, *Alphapolyomavirus castoris*, *Alphapolyomavirus epserotini*, *Alphapolyomavirus myodaubentonii*, *Amphintovirales*, *Anopheles gambiae Moose virus*, *Antheraea semotivirus Tamy*, *Aquambidensovirus asteroid10*, *Aquambidensovirus asteroid11*, *Aquambidensovirus asteroid12*, *Aquambidensovirus asteroid13*, *Aquambidensovirus asteroid14*, *Aquambidensovirus asteroid15*, *Aquambidensovirus asteroid16*, *Aquambidensovirus asteroid17*, *Aquambidensovirus asteroid18*, *Aquambidensovirus asteroid19*, *Aquambidensovirus asteroid20*, *Aquambidensovirus asteroid21*, *Aquambidensovirus asteroid22*, *Aquambidensovirus asteroid3*, *Aquambidensovirus asteroid4*, *Aquambidensovirus asteroid5*, *Aquambidensovirus asteroid6*, *Aquambidensovirus asteroid7*, *Aquambidensovirus asteroid8*, *Aquambidensovirus asteroid9*, *Aquintoviricetes*, *Archintovirales*, *Ascaris lumbricoides Tas virus*, *Asfivirus haemorrhagiae*, *Aveparvovirus anseriform1*, *Aveparvovirus avian1*, *Aveparvovirus galliform2*, *Aveparvovirus passeriform2*, *Aveparvovirus passeriform3*, *Aveparvovirus psittacine1*, *Aveparvovirus psittacine2*, *Aviadenovirus cerasi*, *Aviadenovirus orioli*, *Aviadenovirus oti*, *Aviadenovirus phalacrocoracidae*, *Aviadenovirus roseae*, *Ayintorquevirus*, *Ayintorquevirus ursid28*, *Bamfordvirae*, *Barthadenovirus gerygones*, *Barthadenovirus varani*, *Barthadenovirus zootherae*, *Barthadenovirus zootocae*, *Belvinaviricetes*, *Betabaculovirus psincretae*, *Betafilamentovirus*, *Betafilamentovirus altercocongregatae*, *Betafilamentovirus cocongregatae*, *Betapolyomavirus hipposideri*, *Blattambidensovirus incertum5*, *Bocaparvovirus chiropteran6*, *Bocaparvovirus incertum2*, *Bocaparvovirus incertum3*, *Bocaparvovirus incertum4*, *Bocaparvovirus ungulate10*, *Bombyx mori Pao virus*, *Caenorhabditis elegans Cer13 virus*, *Cardeaviricetes*, *Circovirus baizhenhe*, *Circovirus dever*, *Circovirus patkany*, *Circovirus python*, *Circovirus razbora*, *Commensaviricota*, *Dalettorquevirus*, *Dependoparvovirus anseriform2*, *Dependoparvovirus anseriform3*, *Dependoparvovirus anseriform4*, *Dependoparvovirus anseriform5*, *Dependoparvovirus carnivoran2*, *Dependoparvovirus carnivoran3*, *Dependoparvovirus carnivoran4*, *Dependoparvovirus carnivoran5*, *Dependoparvovirus carnivoran6*, *Dependoparvovirus passeriform1*, *Dependoparvovirus passeriform2*, *Dependoparvovirus rodent3*, *Dependoparvovirus rodent4*, *Drosophila melanogaster Bel virus*, *Drosophila melanogaster Roo virus*, *Drosophila semotivirus Max*, *Drosophila simulans Ninja virus*, *Etatorquevirus felid17*, *Etatorquevirus felid18*, *Etatorquevirus felid19*, *Etatorquevirus felid20*, *Etatorquevirus felid21*, *Etatorquevirus felid22*, *Etatorquevirus felid24*, *Etatorquevirus felid25*, *Etatorquevirus felid26*, *Etatorquevirus felid27*, *Etatorquevirus felid28*, *Etatorquevirus felid35*, *Eupolintoviridae*, *Filamentoviridae*, *Gimeltorquevirus ursid26*, *Gimeltorquevirus ursid27*, *Gyrovirus anas1*, *Gyrovirus anas2*, *Gyrovirus chauna1*, *Hetorquevirus*, *Iotatorquevirus ursid17*, *Mastadenovirus arvicolinae*, *Mastadenovirus capreoli*, *Mastadenovirus cardiodermatis*, *Mastadenovirus desmodi*, *Mastadenovirus fructus*, *Mastadenovirus marmotae*, *Mastadenovirus vespertilionis*, *Monodnaviria*, *Mriyaviricetes*, *Nucleocytoviricota*, *Omicrontorquevirus ursid16*, *Petorquevirus*, *Petorquevirus canid1*, *Petorquevirus ixodi1*, *Petorquevirus viver4*, *Pharingeaviricetes*, *Phypoliviridae*, *Pitorquevirus ursid18*, *Pitorquevirus ursid19*, *Pitorquevirus ursid20*, *Pitorquevirus ursid21*, *Pitorquevirus ursid22*, *Pitorquevirus ursid23*, *Pitorquevirus ursid24*, *Pitorquevirus ursid25*, *Pitorquevirus ursid29*, *Polisuviricotina*, *Preplasmiviricota*, *Prepoliviricotina*, *Produgelaviricota*, *Protoambidensovirus incertum2*, *Protoambidensovirus incertum3*, *Protoambidensovirus incertum4*, *Protoambidensovirus incertum5*, *Protoparvovirus carnivoran6*, *Protoparvovirus carnivoran7*, *Protoparvovirus carnivoran8*, *Protoparvovirus chiropteran2*, *Qoptorquevirus*, *Qoptorquevirus delphin1*, *Qoptorquevirus delphin10*, *Qoptorquevirus delphin11*, *Qoptorquevirus delphin12*, *Qoptorquevirus delphin13*, *Qoptorquevirus delphin14*, *Qoptorquevirus delphin15*, *Qoptorquevirus delphin16*, *Qoptorquevirus delphin17*, *Qoptorquevirus delphin18*, *Qoptorquevirus delphin19*, *Qoptorquevirus delphin2*, *Qoptorquevirus delphin20*, *Qoptorquevirus delphin21*, *Qoptorquevirus delphin22*, *Qoptorquevirus delphin3*, *Qoptorquevirus delphin4*, *Qoptorquevirus delphin5*, *Qoptorquevirus delphin6*, *Qoptorquevirus delphin7*, *Qoptorquevirus delphin8*, *Qoptorquevirus delphin9*, *Rhotorquevirus rodfelid1*, *Sadetorquevirus*, *Sadetorquevirus hominid7*, *Sadetorquevirus hominid8*, *Sanitavirales*, *Schistosoma semotivirus Sinbad*, *Scindoambidensovirus dipteran1*, *Semotivirus beldrosophilae*, *Semotivirus certredecimum*, *Semotivirus maxdrosophilae*, *Semotivirus mooseanophelae*, *Semotivirus ninjadrosophilae*, *Semotivirus paobombycis*, *Semotivirus roodrosophilae*, *Semotivirus sinbadschistosomae*, *Semotivirus suzutakifugu*, *Semotivirus tamyantheraeae*, *Semotivirus tasascaridis*, *Singelaviria*, *Takifugu rubripes Suzu virus*, *Tetrivirus*, *Tetrivirus crimaeaense*, *Thetatorquevirus*, *Thetatorquevirus ursid14*, *Thetatorquevirus ursid15*, *Upsilontorquevirus*, *Upsilontorquevirus ursid6*, *Varidnaviria*, *Virophaviricetes*, *Zayintorquevirus felid10*, *Zayintorquevirus felid11*, *Zayintorquevirus felid12*, *Zayintorquevirus felid13*, *Zayintorquevirus felid14*, *Zayintorquevirus felid15*, *Zayintorquevirus felid16*, *Zayintorquevirus felid29*, *Zayintorquevirus felid30*, *Zayintorquevirus felid31*, *Zayintorquevirus felid32*, *Zayintorquevirus felid33*, *Zayintorquevirus felid34*, *Zayintorquevirus felid7*, *Zayintorquevirus felid8*, *Zayintorquevirus felid9*

## Abstract

The International Committee on Taxonomy of Viruses (ICTV) holds a ratification vote annually after review of newly proposed taxa by ICTV Study Groups and members of the virology community. In March 2025, the vote outcome of the 11 proposals within the mandate of the Animal DNA Viruses and Retroviruses Subcommittee was made public. Here, we provide a summary of the newly accepted proposals. These include reorganization of taxa in the realm *Varidnaviria*, classification of the ‘polinton-like’ viruses into a new family (*Phypoliviridae*) within a new order *Archintovirales*; establishment of a new phylum (*Commensaviricota*) in the kingdom *Shotokuvirae*; the establishment of a new family called *Filamentoviridae* with two new genera and three new species; the addition of four new genera in the family *Anelloviridae* with 70 new species; and the addition of 85 new species in the families *Adenoviridae* (*n*=16), *Baculoviridae* (*n*=5), *Circoviridae* (*n*=5), *Parvoviridae* (*n*=55) and *Polyomaviridae* (*n*=4). Also, in the family *Belpaoviridae*, 11 species were renamed to comply with the binomial requirement for species names.

## Introduction

The Animal DNA Viruses and Retroviruses Subcommittee of the International Committee on Taxonomy of Viruses (ICTV) [1] includes Study Groups for the families *Adenoviridae*, *Anelloviridae*, *Ascoviridae*, *Asfarviridae*, *Baculoviridae, Nudiviridae*, *Bidnaviridae*, *Circoviridae*, *Hepadnaviridae*, *Herpesvirales*, *Hytrosaviridae*, *Iridoviridae*, *Nimaviridae*, *Papillomaviridae*, *Parvoviridae*, *Polydnaviriformidae*, *Polyomaviridae*, *Poxviridae* and *Retroviridae*. These Study Groups include virologists who are experts in viruses classified within these families. As with the ICTV Executive Committee, the 165 members of the Animal DNA Viruses and Retroviruses Subcommittee volunteer their time to advance virus taxonomy [1]. The classified viruses and viriforms in the families represented by the Animal DNA Viruses and Retroviruses Subcommittee are classified into 15 hierarchical ranks for virus classification [2].

Taking various aspects of virus taxonomy into account [2,5], coupled with the binomial species nomenclature (genus +epithet) [6,8], 13 proposals were submitted as part of the Animal DNA Viruses and Retroviruses Subcommittee in 2024; and 11 were accepted by the ICTV Executive Committee for ratification by the broader ICTV membership. Here, we summarize the changes occurring as a result of the ratification of these 11 proposals.

A significant change is the reorganization of taxa in the realm *Varidnaviria*, including movement of the kingdom *Helvetiavirae* into a new realm named *Singelaviria* based on the independent origins of the major capsid proteins encoded by the viruses in the kingdoms *Helvetiavirae* and *Bamfordvirae* [9,10]. Also, as part of this reorganization, a new kingdom named *Abadenavirae* was established as a result of the evolutionary analysis of the protein-primed family B DNA polymerase or its derivatives encoded by tectivirids [11,12], adenovirids [13], adintovirids [14], maveriviricetes and previously unclassified ‘polinton-like’ viruses [11]. The kingdom *Abadenavirae* now includes all bacterial and archaeal viruses with double jelly-roll major capsid proteins, with the exception of tectivirids, which remain in the kingdom *Bamfordvirae* along with all the evolutionarily related eukaryotic viruses. The phylum *Preplasmiviricota* also underwent notable refinements with the establishment of two new subphyla, two new orders and one new family. The previously unassigned family *Yaraviridae* [15] was moved into a new class *Mriyaviricetes* in the phylum *Nucleocytoviricota* [3,16]; the family *Adintoviridae* was renamed *Eupolintoviridae*; the first representative of an extensive group of viruses, broadly known as ‘polinton-like’ viruses [17,18], was classified into a new family, *Phypoliviridae*, within a new order *Archintovirales* [9]. Within the realm *Varidnaviria*, other changes include renaming the species *African swine fever virus* in the family *Asfarviridae* [19] to *Asfivirus haemorrhagiae* to comply with the binomial name rule [6,7] and establishing 16 new species in the family *Adenoviridae* [13].

*Anelloviridae* [20,21] was the only family of eukaryotic single-stranded DNA viruses not assigned to the realm *Monodnaviria* [3]. Although anellovirids do not encode a homologue of the replication-associated endonuclease of the HUH superfamily, the signature of *Monodnaviria*, recent results have shown that they encode capsid protein orthologs with a jelly-roll fold typical of cressdnaviricot capsid proteins, establishing an evolutionary link to other eukaryotic ssDNA viruses, specifically, circovirids [22]. Thus, the family *Anelloviridae* is now classified in the order *Sanitavirales*, class *Cardeaviricetes*, phylum *Commensaviricota*, kingdom *Shotokuvirae* and realm *Monodnaviria*. Furthermore, 4 new genera and 70 new species have been established in the family *Anelloviridae* to classify anellovirids with diverse hosts ranging from marine mammals to terrestrial mammals and avians of various species [22,28]. Other changes within the phylum *Cossaviricota* in the kingdom *Shotokuvirae* include the establishment of 55 new species in the family *Parvoviridae* (order *Piccovirales*, class *Quintoviricetes*) [29] and four species in the family *Polyomaviridae* (order *Sepolyvirales*, class *Papovaviricetes*) [30]. In the phylum *Cressdnaviricota*, five new species were established in the family *Circoviridae* (order *Cirlivirales*, class *Arfiviricetes*) [31].

The family *Filamentoviridae* (order *Lefavirales*, class *Naldaviricetes*), with two genera (*Alphafilamentovirus* and *Betafilamentovirus*) and three species, was established to classify filamentous DNA viruses identified in insects [32,37]. In addition, in the order *Lefavirales* [38], class *Naldaviricetes*, five new species were established in the family *Baculoviridae* [39], and one species (*Alphabaculovirus altermaconfiguratae*) was abolished.

Within the realm *Riboviria*, in the family *Belpaoviridae* (order *Ortervirales*, class *Revtraviricetes*, phylum *Artverviricota*, kingdom *Pararnavirae*) [40], all 11 species were renamed to comply with binomial name rules.

A file including all the Tables of taxonomic changes below is available as a supplementary file to this article.

## Main text

## Contents

2024.001D.Alphabaculovirus_1nsp
2024.002D.Circoviridae_5ns
2024.003D.Polyomaviridae_4ns
2024.004D.Adenoviridae_16ns
2024.005D.Baculoviridae_4nsp_1absp
2024.007D.Filamentoviridae_1nf_2ngen_3nsp
2024.008D.Parvoviridae_55nsp
2024.009D.Anelloviridae_4ngen_70nsp
2024.010D.Varidnaviria_reorg
2024.012D.Shotokuvirae_newphylum
2024.013D.Belpaoviridae_spren

## 2024.001D.Alphabaculovirus_1nsp

**Title**: Create the new species *Alphabaculovirus alterhycuneae* in the genus *Alphabaculovirus* (*Lefavirales: Baculoviridae*)

**Authors**: Peng X-W, Lei C-F, Hu J, Sun XL (sunxl@wh.iov.cn)

**Summary**

**Taxonomic rank(s) affected**
Species
**Description of current taxonomy**
In the genus *Alphabaculovirus* (family *Baculoviridae*), there are 65 species.
**Proposed taxonomic change(s)**
Add one (1) new species to genus *Alphabaculovirus*.
**Justification**
The genome of the virus Hypantria cuneae nulceopolyhedrovirus B was fully sequenced using a high-throughput method. The divergence in the phylogenetic tree and the K2P distances based on the 38 core-gene concatenated alignment revealed that this virus belongs to a novel species of *Alphabaculovirus*. For this new species, the species name *Alphabaculovirus alterhycuneae* is suggested, following the binomial naming proposal as submitted in 2022 and ratified by the ICTV in April 2023 [41].
**Submitted**: 05/04/23

**Table 1.** *Alphabaculovirus*, 1 new taxon*

**Table:** 

Operation	Rank	New taxon name	Exemplar	Accession
New taxon	Species	*Alphabaculovirus alterhycuneae*	Hypantria cunea nucleopolyhedrovirus B	OL686893

*Source/full text:https://ictv.global/ictv/proposals/2024.001D.Alphabaculovirus_1nsp.zip.

## 2024.002D.Circoviridae_5ns

**Title**: Create five new species in the genus *Circovirus* (*Cirlivirales: Circoviridae*)

**Authors**: Tarján ZL (tarjan.zoltan@vmri.hun-ren.hu), Benkő M, Egyed L, Harrach B

**Summary**

**Taxonomic rank(s) affected**
Species
**Description of current taxonomy**
One hundred and fifty-five species (65 circoviruses and 90 cycloviruses) in two genera within the family *Circoviridae*.
**Proposed taxonomic change(s)**
Add five (5) new species to the genus *Circovirus*.
**Justification**
Based on genome organization and phylogenetic analyses, the establishment of five new species in the genus *Circovirus* is proposed. The species demarcation is based on the genome-wide pairwise identity between circovirids (less than 80% identity as established species demarcation criterion) [31,42].
**Submitted**: 21/06/24

**Table 2.** *Circoviridae*, 5 new taxa*

**Table:** 

Operation	Rank	New taxon name	Exemplar	Accession
New taxon	Species	*Circovirus dever*	bream circovirus 1	KF358279
New taxon	Species	*Circovirus razbora*	Pseudorasbora circovirus 1	MN837844
New taxon	Species	*Circovirus baizhenhe*	white-naped crane circovirus 1	MN928908
New taxon	Species	*Circovirus patkany*	brown rat circovirus 1	OR553090
New taxon	Species	*Circovirus python*	black-headed python circovirus 1	MH368042

*Source/full text: https://ictv.global/ictv/proposals/2024.002D.Circoviridae_5ns.zip.

## 2024.003D.Polyomaviridae_4ns

**Title**: Create four new species in the genera *Alphapolyomavirus* and *Betapolyomavirus* (*Polyomaviridae*)

**Authors**: Surján A (surjan.andras@vmri.hun-ren.hu), Vidovszky MZ, Postler TS, Harrach B,

**Summary**

**Taxonomic rank(s) affected**
Species
**Description of current taxonomy**
One hundred and eighteen species in eight genera in the family *Polyomaviridae*.
**Proposed taxonomic change(s)**
Add four (4) new species, three (3) to the genus *Alphapolyomavirus* and one (1) to the genus *Betapolyomavirus*
**Justification**
Novel polyomaviruses have been detected in bat guano and Eurasian beaver kidney tissue samples. Three novel bat polyomaviruses and the beaver polyomavirus meet the criteria of establishing a new species. The phylogenetic distance of their Large T-antigen nucleotide sequences is more than 15% to members of accepted polyomavirus species, and they originate from new hosts.
**Submitted**: 21/06/24

**Table 3.** *Polyomaviridae*, 4 new taxa*

**Table:** 

Operation	Rank	New taxon name	Exemplar	Accession
New taxon	Species	*Alphapolyomavirus castoris*	Castor fiber polyomavirus 1	OR735477
New taxon	Species	*Alphapolyomavirus epserotini*	Eptesicus serotinus polyomavirus 1	OK428546
New taxon	Species	*Alphapolyomavirus myodaubentonii*	Myotis daubentonii polyomavirus 2	OK300052
New taxon	Species	*Betapolyomavirus hipposideri*	Rhinolophus hipposideros polyomavirus 1	MT276890

*Source/full text: https://ictv.global/ictv/proposals/2024.003D.Polyomaviridae_4ns.zip.

## 2024.004D.Adenoviridae_16ns

**Title**: Create 16 new species in the genera *Aviadenovirus, Barthadenovirus* and *Mastadenovirus* (*Rowavirales: Adenoviridae*)

**Authors**: Benkő M, Arnberg N, Hess M, Kaján GL, Kajon A, Mittal SK, Podgorski II, Postler TS, San Martín C, Wadell G, Watanabe H, Harrach B (harrach.balazs@vmri.hun-ren.hu).

**Summary**

**Taxonomic rank(s) affected**
Species
**Description of current taxonomy**
One hundred and nine species in six genera in the family *Adenoviridae*.
**Proposed taxonomic change(s)**
Add 16 novel species: seven (7) to the genus *Mastadenovirus,* five (5) to the genus *Aviadenovirus* and four (4) to the genus *Barthadenovirus*.
**Justification**
Novel adenovirus sequences have been submitted to GenBank (many from metagenomic data) reflecting very rich diversity (https://sites.google.com/site/adenoseq). From these sequences, 16 full-length or almost full-length (coding-complete) animal adenovirus genomes originating from 7 mammal, 7 bird and 2 reptilian species merit the establishment of new species. The phylogenetic distance of their DNA polymerase amino acid sequences is more than 15% to members of accepted adenovirus species (this is the main demarcation criterion). Furthermore, they originate from new hosts or from hosts different from those of existing species, and/or have a characteristic whole-genome GC content percentage difference.
**Submitted**: 21/06/24

**Table 4.** *Adenoviridae*, 16 new taxa*

**Table:** 

Operation	Rank	New taxon name	Exemplar	Accession
New taxon	Species	*Mastadenovirus marmotae*	marmot adenovirus 1	PP098964
New taxon	Species	*Mastadenovirus capreoli*	roe deer adenovirus 1; adenovirus capreolus32301	BK066828
New taxon	Species	*Mastadenovirus vespertilionis*	bat adenovirus 33390	BK066631
New taxon	Species	*Mastadenovirus desmodi*	vampire bat adenovirus; adenovirus desmodus35011	BK066905
New taxon	Species	*Mastadenovirus cardiodermatis*	heart-nosed bat adenovirus	PP711818
New taxon	Species	*Mastadenovirus fructus*	Leschenault's rousette adenovirus	OR998962
New taxon	Species	*Mastadenovirus arvicolinae*	vole adenovirus 1; adenovirus myodes38640	BK066403
New taxon	Species	*Aviadenovirus phalacrocoracidae*	great cormorant adenovirus 1	OR529407
New taxon	Species	*Aviadenovirus oti*	Eurasian scops owl adenovirus 1; Otus scops adenovirus	ON843719
New taxon	Species	*Aviadenovirus orioli*	black-naped oriole adenovirus; Oriolus adenovirus	MZ819701
New taxon	Species	*Aviadenovirus roseae*	psittacine adenovirus 12	OR871655
New taxon	Species	*Aviadenovirus cerasi*	duck adenovirus 6	MK757473
New taxon	Species	*Barthadenovirus gerygones*	grey warbler adenovirus 1	OQ986611
New taxon	Species	*Barthadenovirus zootherae*	scaly thrush (Zoothera dauma) adenovirus 1	OR233592
New taxon	Species	*Barthadenovirus varani*	varanus adenovirus 37597	BK066675
New taxon	Species	*Barthadenovirus zootocae*	viviparous lizard adenovirus 1; adenovirus zootoca35082	BK066448

*Source/full text: https://ictv.global/ictv/proposals/2024.004D.Adenoviridae_16ns.zip.

## 2024.005D.Baculoviridae_4nsp_1absp

**Title**: Create four new species and abolish one species in the family *Baculoviridae*

**Authors**: van Oers MM, Abd-Alla AMM, Bateman KS, Bojko J, Harrison RL (robert.l.harrison@usda.gov), Herniou EA, Sun XL, Jehle JA, Krell PJ, Ribeiro BM

**Summary**

**Taxonomic rank(s) affected**
Species
**Description of current taxonomy**
There are currently 65 species in the genus *Alphabaculovirus* and 28 species in the genus *Betabaculovirus* of the family *Baculoviridae*.
**Proposed taxonomic change(s)**
Add three (3) new species in the genus *Alphabaculovirus,* add one (1) new species in the genus *Betabaculovirus,* and abolish one (1) species, *Alphabaculovirus altermaconfiguratae*.
**Justification**
Analysis of recently sequenced baculovirus genomes identified four viruses that each represent a previously undescribed baculovirus species, in accordance with the species demarcation criteria defined for the family *Baculoviridae*. Analysis of the genomes of viruses from the species *Alphabaculovirus mabrassicae, Alphabaculovirus maconfiguratae* and *Alphabaculovirus altermaconfiguratae* indicates that *Alphabaculovirus altermaconfiguratae* is redundant and should be abolished. The creation of *Alphabaculovirus mabrassicae* precedes that of both *Alphabaculovirus maconfiguratae* and *Alphabaculovirus altermaconfiguratae,* and the exemplar isolate *of Alphabaculovirus mabrassicae* falls in the same clade as the exemplar isolate *of Alphabaculovirus altermaconfiguratae,* so we propose to abolish *Alphabaculovirus altermaconfiguratae*.
**Submitted**: 30/04/24

**Table 5.** *Baculoviridae*, 4 new taxa*

**Table:** 

Operation	Rank	New taxon name	Exemplar	Accession
New taxon	Species	*Alphabaculovirus pastagnalis*	Parapoynx stagnalis nucleopolyhedrovirus	ON704650
New taxon	Species	*Alphabaculovirus pavitrealis*	Palpita vitrealis nucleopolyhedrovirus	OL685370
New taxon	Species	*Alphabaculovirus spocosmioidis*	Spodoptera cosmioides nucleopolyhedrovirus	MK419955
New taxon	Species	*Betabaculovirus psincretae*	Psilogramma increta granulovirus	ON803509

**Table 6.** *Baculoviridae*, 1 abolish taxon*

**Table:** 

Operation	Rank	Abolished taxon name
Abolish taxon	Species	*Alphabaculovirus altermaconfiguratae*

*Source/full text: https://ictv.global/ictv/proposals/2024.005D.Baculoviridae_4nsp_1absp.zip.

## 2024.007D.Filamentoviridae_1nf_2ngen_3nsp

**Title**: Create a new virus family in the *Lefavirales* order named *Filamentoviridae* with two genera *Alphafilamentovirus* and *Betafilamentovirus* and three species.

**Authors**: Bézier A (annie.bezier@univ-tours.fr), Leobold M, Guinet B, Drezen J-M, Herniou EA, Varaldi J

**Summary**

**Taxonomic rank(s) affected**
Establishment of a new highly diverse viral family within the order *Lefavirales* in the class *Naldaviricetes,* the *Filamentoviridae,* comprising two genera: *Alphafilamentovirus,* with the species *Alphafilamentovirus leboulardi,* and *Betafilamentovirus,* with the species *Betafilamentovirus cocongregatae* and *Betafilamentovirus altercocongregatae*.
**Description of current taxonomy**
The class *Naldaviricetes* currently includes four families: *Baculoviridae, Nudiviridae, Hytrosaviridae* and *Nimaviridae,* the first three belonging to the order of *Lefavirales*.
**Proposed taxonomic change(s)**
Create *Filamentoviridae,* a new family in the order *Lefavirales* within the class *Naldaviricetes,* with two (2) genera (*Alphafilamentovirus* and *Betafilamentovirus)* and three species.
**Justification**
New large arthropod-specific dsDNA viruses, which have been described as filamentous particles since the 1970s, have recently been characterized at the genomic level [33]. These viruses share signatures of members of the class *Naldaviricetes* and order *Lefavirales,* while encoding specific core genes that distinguish them from the established families of this order. Phylogenetic tree reconstruction indicates that these filamentous viruses form a monophyletic clade distinct from that of their closest relatives, *Hytrosaviridae,* and supports the creation of a new family, that we propose to name *Filamentoviridae*. These viruses appear to be preferentially associated with hymenopteran insects with a parasitoid lifestyle [33]. The effects of filamentous viruses on their hosts are still poorly understood compared with other members of the *Naldaviricetes*. So far, only the Leptopilina boulardi filamentous virus has been studied for its effect and is described as inducing a behavioural manipulation of wasp oviposition decisions and benefiting from the vertical and horizontal transmission.
**Submitted**: 04/06/24; **Revised**: 23/10/24

**Table 7.** *Filamentoviridae*, 6 new taxa*

**Table:** 

Operation	Rank	New taxon name	Exemplar	Accession
New taxon	Family	*Filamentoviridae*		
New taxon	Genus	*Alphafilamentovirus*		
New taxon	Species	*Alphafilamentovirus leboulardi*	Leptopilina boulardi filamentous virus	KY009685
New taxon	Genus	*Betafilamentovirus*		
New taxon	Species	*Betafilamentovirus cocongregatae*	Cotesia congregata filamentous virus 1	OY734801
New taxon	Species	*Betafilamentovirus altercocongregatae*	Cotesia congregata filamentous virus 2	OR120048

*Source/full text: https://ictv.global/ictv/proposals/2024.007D.Filamentoviridae_1nf_2ngen_3nsp.zip.

## 2024.008D.Parvoviridae_55nsp

**Title:** Creating 55 new species in family *Parvoviridae*

**Authors**: Pénzes J (Judycash08@gmail.com), Canuti M, François S, Söderlund-Venermo M

**Summary**

**Taxonomic rank(s) affected**
Subfamily *Densovirinae,* genera *Blattambidensovirus, Scindoambidensovirus, Protoambidensovirus, Aquambidensovirus*; subfamily *Parvovirinae,* genera *Aveparvovirus, Bocaparvovirus, Dependoparvovirus, Protoparvovirus*
**Description of current taxonomy**
The family currently includes:
Subfamily *Densovirinae* with 11 genera and 38 species
Subfamily *Parvovirinae* with 11 genera and 107 species
Subfamily *Hamaparvovirinae* with 5 genera and 42 species
Unassigned genus *Metalloincertoparvovirus* with one species
**Proposed taxonomic change(s)**
This taxonomic proposal describes the creation of 26 new species in the subfamily *Densovirinae* and of 29 new species in the subfamily *Parvovirinae*. Additionally, the virus sequence derived from sequencing approach criteria was modified to allow for the classification of sequences derived from cDNA-based metatranscriptomes if specific criteria are met.
**Justification**
Several novel parvoviruses have been described in the literature that fulfil the criteria to be classified as separate species. Additionally, various coding-complete genomes derived from metatranscriptomic experiments have been published, and the virus definition was changed to allow the classification of these viruses if there are reasons to believe that the sequences originate from viral DNA, i.e. the sample preparation did not involve a DNase treatment step.
**Submitted**: 08/06/24

**Table 8.** *Parvoviridae*, 55 new taxa*. Table too large, see supplementary information sheetsupp_info_tab_8

*Source/full text: https://ictv.global/ictv/proposals/2024.008D.Parvoviridae_55nsp.zip.

## 2024.009D.Anelloviridae_4ngen_70nsp

**Title:** Establish 4 new genera, 70 new species and abolish 1 genus in the family *Anelloviridae*

**Authors:** Kraberger S (simona.kraberger@asu.edu), Opriessnig T, Maggi F, Celer V, Okamoto H, Biagini P, Krupovic M, Varsani A

**Summary**

**Taxonomic rank(s) affected**
Genus, species
**Description of current taxonomy**
The family *Anelloviridae* currently includes 34 genera and 173 species [20]. Over the last few years, numerous diverse anelloviruses have been identified in various animals. Classification is based on the species demarcation criteria of 69% ORF1 nucleotide pairwise identity and phylogenetic analyses [20].
**Proposed taxonomic change(s)**
Add 70 new species to accommodate the unclassified anelloviruses and add four (4) new genera.
**Justification**
Here, we update the current anellovirus taxonomy by undertaking an analysis of anelloviruses whose full genome sequences have been determined. These changes are based on the species demarcation criteria of 69% ORF1 nucleotide pairwise identity and updated phylogenetic analyses of the ORF1 protein sequences.
**Submitted**: 14/06/24; **Revised**: 04/10/24

**Table 9.** *Anelloviridae*, 74 new taxa*. Table too large, see supplementary information sheetsupp_info_tab_9

**Table 10.** *Anelloviridae*, 6 move; rename taxa*

**Table:** 

Operation	Rank	New taxon name	Old parent taxon	New parent taxon	Old taxon name
Move; rename taxon	Species	*Upsilontorquevirus ursid6*	*Dalettorquevirus*	*Upsilontorquevirus*	*Dalettorquevirus ursid6*
Move; rename taxon	Species	*Sadetorquevirus hominid8*	*Hetorquevirus*	*Sadetorquevirus*	*Hetorquevirus hominid8*
Move; rename taxon	Species	*Sadetorquevirus hominid7*	*Hetorquevirus*	*Sadetorquevirus*	*Hetorquevirus hominid7*
Move; rename taxon	Species	*Petorquevirus ixodi1*	*Thetatorquevirus*	*Petorquevirus*	*Thetatorquevirus ixodi1*
Move; rename taxon	Species	*Petorquevirus canid1*	*Thetatorquevirus*	*Petorquevirus*	*Thetatorquevirus canid1*
Move; rename taxon	Species	*Petorquevirus viver4*	*Thetatorquevirus*	*Petorquevirus*	*Thetatorquevirus viver4*

**Table 11.** *Anelloviridae*, 1 abolish taxon*

**Table:** 

Operation	Rank	Abolished taxon name
Abolish taxon	Genus	*Dalettorquevirus*

*Source/full text: https://ictv.global/ictv/proposals/2024.009D.Anelloviridae_4ngen_70nsp.zip.

## 2024.010D.Varidnaviria_reorg

**Title:** Reorganization of the realm *Varidnaviria*

**Authors:** Koonin EV (koonin@ncbi.nlm.nih.gov), Fischer MG, Yutin N, Kuhn JH, Krupovic M (mart.krupovic@pasteur.fr).

**Summary**

**Taxonomic rank(s) affected**
Realm (*Varidnaviria*)
**Description of current taxonomy**
The realm currently includes two kingdoms: *Bamfordvirae* (two phyla with a total of six classes and one unassigned family) and *Helvetiavirae* (one phylum including one class)
**Proposed taxonomic change(s)**
Create a new realm to accommodate *Helvetiavirae;* create a new varidnavirian kingdom to accommodate five (5) previously bamfordviraen orders; create two (2) subphyla in the bamfordviraen phylum *Preplasmiviricota,* assigning *Tectiliviricetes* to one and the remaining taxa to the other, which is also expanded by three (3) new classes to accommodate polinton-like viruses and *Adenoviridae*.
**Justification**
A thorough genomic and proteomic analysis revealed previously unrecognized evolutionary relationships among the various varidnaviraen taxa.
**Submitted**: 21/06/24; **Revised**: 04/10/24

**Table 12.** *Varidnaviria*, 13 new taxa*

**Table:** 

Operation	Rank	New taxon name	Exemplar	Accession
New taxon	Realm	*Singelaviria*		
New taxon	Kingdom	*Abadenavirae*		
New taxon	Phylum	*Produgelaviricota*		
New taxon	Class	*Belvinaviricetes*		
New taxon	Subphylum	*Prepoliviricotina*		
New taxon	Subphylum	*Polisuviricotina*		
New taxon	Class	*Pharingeaviricetes*		
New taxon	Class	*Aquintoviricetes*		
New taxon	Order	*Archintovirales*		
New taxon	Family	*Phypoliviridae*		
New taxon	Genus	*Tetrivirus*		
New taxon	Species	*Tetrivirus crimaeaense*	Tetraselmis viridis virus S1	HQ332143
New taxon	Class	*Mriyaviricetes*		

**Table 13.** *Varidnaviria*, 11 move taxa*

**Table:** 

Operation	Rank	Taxon name	Old parent taxon	New parent taxon
Move taxon	Kingdom	*Helvetiavirae*	*Varidnaviria*	*Singelaviria*
Move taxon	Class	*Ainoaviricetes*	*Preplasmiviricota*	*Produgelaviricota*
Move taxon	Order	*Atroposvirales*	*Tectiliviricetes*	*Belvinaviricetes*
Move taxon	Order	*Belfryvirales*	*Tectiliviricetes*	*Belvinaviricetes*
Move taxon	Order	*Coyopavirales*	*Tectiliviricetes*	*Belvinaviricetes*
Move taxon	Order	*Vinavirales*	*Tectiliviricetes*	*Belvinaviricetes*
Move taxon	Family	*Autolykiviridae*	*Tectiliviricetes*	*Vinavirales*
Move taxon	Class	*Tectiliviricetes*	*Preplasmiviricota*	*Prepoliviricotina*
Move taxon	Order	*Rowavirales*	*Tectiliviricetes*	*Pharingeaviricetes*
Move taxon	Class	*Polintoviricetes*	*Preplasmiviricota*	*Polisuviricotina*
Move taxon	Family	*Yaraviridae*	*Bamfordvirae*	*Mriyaviricetes*

**Table 14.** *Varidnaviria*, 3 move; rename taxa*

**Table:** 

Operation	Rank	New taxon name	Old parent taxon	New parent taxon	Old taxon name
Move; rename taxon	Class	*Virophaviricetes*	*Preplasmiviricota*	*Polisuviricotina*	*Maveriviricetes*
Move; rename taxon	Family	*Eupolintoviridae*	*Orthopolintovirales*	*Amphintovirales*	*Adintoviridae*
Move; rename taxon	Order	*Amphintovirales*	*Polintoviricetes*	*Polintoviricetes*	*Orthopolintovirales*

**Table 15.** *Varidnaviria*, 1 rename taxon*

**Table:** 

Operation	Rank	New taxon name	Previous taxon name
Rename taxon	Species	*Asfivirus haemorrhagiae*	*African swine fever virus*

*Source/full text: https://ictv.global/ictv/proposals/2024.010D.Varidnaviria_reorg.zip.

## 2024.012D.Shotokuvirae_newphylum

**Title:** Create a new phylum *Commensaviricota* for the kingdom *Shotokuvirae* and the family *Anelloviridae*

**Authors:** Varsani A, Butkovic A, Kraberger S, Koonin EV, Krupovic M (mart.krupovic@pasteur.fr).

**Summary**

**Taxonomic rank(s) affected**
*Monodnaviria*, *Shotokuvirae*
**Description of current taxonomy**
The kingdom *Shotokuvirae* includes two (2) phyla, for eukaryotic ssDNA and related dsDNA viruses classified into the phyla *Cressdnaviricota* and *Cossaviricota,* respectively. *Anelloviridae* is the only family of eukaryotic ssDNA viruses not assigned to the realm *Monodnaviria*.
**Proposed taxonomic change(s)**
Move the family *Anelloviridae* into a new order, within a new class and a new phylum *Commensaviricota* within the kingdom *Shotokuvirae*. The intermediate taxa between the phylum and family will be the order *Sanitavirales* and the class *Cardeaviricetes*.
**Justification**
Sequence and structural comparisons suggest that anelloviruses evolved from a circovirus-like ancestor through gradual augmentation of the capsid protein and loss of the Rep protein genes.
**Submitted**: 24/06/24

**Table 16.** *Shotokuvirae*, 3 new taxa*

**Table:** 

Operation	Rank	New taxon name
New taxon	Phylum	*Commensaviricota*
New taxon	Class	*Cardeaviricetes*
New taxon	Order	*Sanitavirales*

**Table 17.** *Shotokuvirae*, 1 move taxon*

**Table:** 

Operation	Rank	Taxon name	Old parent taxon	New parent taxon
Move taxon	Family	*Anelloviridae*		*Sanitavirales*

*Source/full text: https://ictv.global/ictv/proposals/2024.012D.Shotokuvirae_newphylum.zip.

## 2024.013D.Belpaoviridae_spren

**Title:** Rename all species to conform with the ICTV-mandated binomial format (*Ortervirales: Belpaoviridae*)

**Authors:** Krupovic M (mart.krupovic@pasteur.fr), Kuhn JH

**Summary**

**Taxonomic rank(s) affected**
Species
**Description of current taxonomy**
*Belpaoviridae: Semotivirus* (11 species).
**Proposed taxonomic change(s)**
Rename all belpaovirid/semotivirus species to conform with the ICTV-mandated binomial format.
**Justification**
Species in the family *Belpaoviridae* do not conform with the ICTV-mandated binomial format.
**Submitted**: 21/06/24

**Table 18.** *Belpaoviridae*, 11 rename taxa*

**Table:** 

Operation	Rank	New taxon name	Previous taxon name
Rename taxon	Species	*Semotivirus mooseanophelae*	*Anopheles gambiae Moose virus*
Rename taxon	Species	*Semotivirus tamyantheraeae*	*Antheraea semotivirus Tamy*
Rename taxon	Species	*Semotivirus tasascaridis*	*Ascaris lumbricoides Tas virus*
Rename taxon	Species	*Semotivirus paobombycis*	*Bombyx mori Pao virus*
Rename taxon	Species	*Semotivirus certredecimum*	*Caenorhabditis elegans Cer13 virus*
Rename taxon	Species	*Semotivirus beldrosophilae*	*Drosophila melanogaster Bel virus*
Rename taxon	Species	*Semotivirus roodrosophilae*	*Drosophila melanogaster Roo virus*
Rename taxon	Species	*Semotivirus maxdrosophilae*	*Drosophila semotivirus Max*
Rename taxon	Species	*Semotivirus ninjadrosophilae*	*Drosophila simulans Ninja virus*
Rename taxon	Species	*Semotivirus sinbadschistosomae*	*Schistosoma semotivirus Sinbad*
Rename taxon	Species	*Semotivirus suzutakifugu*	*Takifugu rubripes Suzu virus*

*Source/full text: https://ictv.global/ictv/proposals/2024.013D.Belpaoviridae_spren.zip.

## Supplementary material

10.1099/jgv.0.002113 Supplementary Material 1.

## Abbreviations

ICTV: International Committee on Taxonomy of Viruses

## References

[1] Siddell SG, Smith DB, Adriaenssens E, Alfenas-Zerbini P, Dutilh BE (2023). Virus taxonomy and the role of the International Committee on Taxonomy of Viruses (ICTV). J Gen Virol.

[2] International Committee on Taxonomy of Viruses Executive Committee (2020). The new scope of virus taxonomy: partitioning the virosphere into 15 hierarchical ranks. Nat Microbiol.

[3] Koonin EV, Dolja VV, Krupovic M, Varsani A, Wolf YI (2020). Global organization and proposed megataxonomy of the virus world. Microbiol Mol Biol Rev.

[4] Simmonds P, Adams MJ, Benkő M, Breitbart M, Brister JR (2017). Consensus statement: virus taxonomy in the age of metagenomics. Nat Rev Microbiol.

[5] Simmonds P, Adriaenssens EM, Zerbini FM, Abrescia NGA, Aiewsakun P (2023). Four principles to establish a universal virus taxonomy. PLoS Biol.

[6] Siddell SG, Walker PJ, Lefkowitz EJ, Mushegian AR, Dutilh BE (2020). Binomial nomenclature for virus species: a consultation. Arch Virol.

[7] Walker PJ, Siddell SG, Lefkowitz EJ, Mushegian AR, Adriaenssens EM (2021). Changes to virus taxonomy and to the International Code of Virus Classification and Nomenclature ratified by the International Committee on Taxonomy of Viruses (2021). Arch Virol.

[8] Postler TS, Rubino L, Adriaenssens EM, Dutilh BE, Harrach B (2022). Guidance for creating individual and batch latinized binomial virus species names. J Gen Virol.

[9] Koonin EV, Fischer MG, Kuhn JH, Krupovic M (2024). The polinton-like supergroup of viruses: evolution, molecular biology, and taxonomy. Microbiol Mol Biol Rev.

[10] Krupovic M, Makarova KS, Koonin EV (2022). Cellular homologs of the double jelly-roll major capsid proteins clarify the origins of an ancient virus kingdom. Proc Natl Acad Sci U S A.

[11] Krupovic M, Kuhn JH, Fischer MG, Koonin EV (2024). Natural history of eukaryotic DNA viruses with double jelly-roll major capsid proteins. Proc Natl Acad Sci U S A.

[12] Redrejo-Rodríguez M, Ordóñez CD, Berjón-Otero M, Moreno-González J, Aparicio-Maldonado C (2017). Primer-independent DNA synthesis by a family B DNA polymerase from self-replicating mobile genetic elements. Cell Rep.

[13] Benkő M, Aoki K, Arnberg N, Davison AJ, Echavarría M (2022). ICTV Virus Taxonomy Profile: *Adenoviridae* 2022. J Gen Virol.

[14] Starrett GJ, Tisza MJ, Welch NL, Belford AK, Peretti A (2021). Adintoviruses: a proposed animal-tropic family of midsize eukaryotic linear dsDNA (MELD) viruses. Virus Evol.

[15] de Miranda Boratto PV, Oliveira GP, Abrahão JS (2022). “Yaraviridae”: a proposed new family of viruses infecting *Acanthamoeba castellanii*. Arch Virol.

[16] Yutin N, Mutz P, Krupovic M, Koonin EV (2024). Mriyaviruses: small relatives of giant viruses. mBio.

[17] Bellas C, Hackl T, Plakolb MS, Koslová A, Fischer MG (2023). Large-scale invasion of unicellular eukaryotic genomes by integrating DNA viruses. Proc Natl Acad Sci U S A.

[18] Yutin N, Shevchenko S, Kapitonov V, Krupovic M, Koonin EV (2015). A novel group of diverse Polinton-like viruses discovered by metagenome analysis. BMC Biol.

[19] Alonso C, Borca M, Dixon L, Revilla Y, Rodriguez F (2018). ICTV Virus Taxonomy Profile: *Asfarviridae*. J Gen Virol.

[20] Varsani A, Kraberger S, Opriessnig T, Maggi F, Celer V (2023). *Anelloviridae* taxonomy update 2023. Arch Virol.

[21] Varsani A, Opriessnig T, Celer V, Maggi F, Okamoto H (2021). Taxonomic update for mammalian anelloviruses (family *Anelloviridae*). Arch Virol.

[22] Butkovic A, Kraberger S, Smeele Z, Martin DP, Schmidlin K (2023). Evolution of anelloviruses from a circovirus-like ancestor through gradual augmentation of the jelly-roll capsid protein. Virus Evol.

[23] De Koch MD, Krupovic M, Fielding R, Smith K, Schiavone K (2025). Novel lineage of anelloviruses with large genomes identified in dolphins. J Virol.

[24] Kraberger S, Serieys LEK, Richet C, Fountain-Jones NM, Baele G (2021). Complex evolutionary history of felid anelloviruses. Virology.

[25] Liu Y, Sun L, Tu Z, Sun S, Sun Y (2022). Virome profiling of an Amur leopard cat reveals multiple anelloviruses and a bocaparvovirus. Vet Sci.

[26] Zhao M, Yue C, Yang Z, Li Y, Zhang D (2022). Viral metagenomics unveiled extensive communications of viruses within giant pandas and their associated organisms in the same ecosystem. Sci Total Environ.

[27] Goldberg TL, Clyde VL, Gendron-Fitzpatrick A, Sibley SD, Wallace R (2018). Severe neurologic disease and chick mortality in crested screamers (*Chauna torquata*) infected with a novel gyrovirus. Virology.

[28] Cibulski S, Weber MN, de Sales Lima FE, Lima DA de, Fernandes Dos Santos H (2020). Viral metagenomics in Brazilian Pekin ducks identifies two gyrovirus, including a new species, and the potentially pathogenic duck circovirus. Virology.

[29] Cotmore SF, Agbandje-McKenna M, Canuti M, Chiorini JA, Eis-Hubinger A-M (2019). ICTV Virus Taxonomy Profile: *Parvoviridae*. J Gen Virol.

[30] Moens U, Calvignac-Spencer S, Lauber C, Ramqvist T, Feltkamp MCW (2017). ICTV Virus Taxonomy Profile: *Polyomaviridae*. J Gen Virol.

[31] Breitbart M, Delwart E, Rosario K, Segalés J, Varsani A (2017). ICTV Virus Taxonomy Profile: *Circoviridae*. J Gen Virol.

[32] Gauthier J, Boulain H, van Vugt JJFA, Baudry L, Persyn E (2021). Chromosomal scale assembly of parasitic wasp genome reveals symbiotic virus colonization. *Commun Biol*.

[33] Guinet B, Leobold M, Herniou EA, Bloin P, Burlet N (2024). A novel and diverse family of filamentous DNA viruses associated with parasitic wasps. Virus Evol.

[34] Lepetit D, Gillet B, Hughes S, Kraaijeveld K, Varaldi J (2016). Genome sequencing of the behavior manipulating virus LbFV reveals a possible new virus family. Genome Biol Evol.

[35] Wallace MA, Coffman KA, Gilbert C, Ravindran S, Albery GF (2021). The discovery, distribution, and diversity of DNA viruses associated with *Drosophila melanogaster* in Europe. Virus Evol.

[36] Inwood SN, Skelly J, Guhlin JG, Harrop TWR, Goldson SL (2023). Chromosome-level genome assemblies of two parasitoid biocontrol wasps reveal the parthenogenesis mechanism and an associated novel virus. BMC Genom.

[37] Varaldi J, Fouillet P, Ravallec M, López-Ferber M, Boulétreau M (2003). Infectious behavior in a parasitoid. Science.

[38] van Oers MM, Herniou EA, Jehle JA, Krell PJ, Abd-Alla AMM (2023). Developments in the classification and nomenclature of arthropod-infecting large DNA viruses that contain *pif* genes. Arch Virol.

[39] Harrison RL, Herniou EA, Jehle JA, Theilmann DA, Burand JP (2018). ICTV Virus Taxonomy Profile: *Baculoviridae*. J Gen Virol.

[40] Soriano B, Krupovic M, Llorens C (2021). ICTV Virus Taxonomy Profile: *Belpaoviridae* 2021. J Gen Virol.

[41] Zerbini FM, Siddell SG, Lefkowitz EJ, Mushegian AR, Adriaenssens EM (2023). Changes to virus taxonomy and the ICTV Statutes ratified by the International Committee on Taxonomy of Viruses (2023). Arch Virol.

[42] Rosario K, Breitbart M, Harrach B, Segalés J, Delwart E (2017). Revisiting the taxonomy of the family *Circoviridae*: establishment of the genus *Cyclovirus* and removal of the genus *Gyrovirus*. Arch Virol.

